# A Case of Acute Psychosis in an Adolescent Male

**DOI:** 10.1155/2014/937631

**Published:** 2014-03-30

**Authors:** Ghufran Babar, Ramin Alemzadeh

**Affiliations:** ^1^Section of Pediatric Endocrinology, Children's Mercy Hospitals and Clinics, 3101 Broadway Boulevard, Kansas City, MO 64111, USA; ^2^Division of Pediatric Endocrinology, University of Illinois College of Medicine at Chicago, 1853 W. Polk Street, Chicago, IL 60612, USA

## Abstract

Primary hyperparathyroidism (PHPT) is a disorder of calcium homeostasis. We report the case of a 17-year-old adolescent male, who presented with an acute psychosis coinciding with severe hypercalcemia and markedly elevated intact parathyroid hormone (iPTH) level and low vitamin D level. A Sestamibi scan showed a positive signal inferior to the left lobe of the thyroid gland. He had only a partial response to the initial medical and psychiatric management. The enlarged parathyroid gland was resected surgically and postoperatively serum calcium and iPTH levels normalized. The histopathology was compatible with a benign adenoma. Patient's acute psychotic symptoms resolved gradually after surgery; however he remained under psychiatric care for the behavioral issues for about 6 months after surgery. While psychosis is a rare clinical manifestation of hypercalcemia secondary to PHPT in pediatric population, it should be considered as a clinical clue in an otherwise asymptomatic pediatric patient.

## 1. Introduction

Primary hyperparathyroidism (PHPT) is a disorder of calcium homeostasis. It is rare in pediatric population (age < 18 years) and is usually caused by a single parathyroid adenoma (PA) [1–3]. PA has an incidence of 2–5 per 100,000 children [1]. Elevated levels of intact parathyroid hormone (iPTH) lead to persistent hypercalcemia. PA commonly involves the inferior parathyroid glands, although it can be found in several ectopic sites including thyroid gland, thymus, pericardium, and retroesophageal areas [4]. Hypercalcemia due to PA presents initially as nonspecific symptoms like polyuria, weakness, fatigue, irritability, anorexia, abdominal pain, nausea, emesis, and weight loss. Therefore, the diagnosis of hypercalcemia is often delayed due to subtle clinical symptoms until the complications emerge [1]. These include renal stones, osteopenia and osteoporosis, osteitis fibrosa cystica, peptic ulcer disease, pancreatitis, constipation, hypertension, arrhythmias (shortening of QT interval) [5], and neuropsychiatric diseases [6].

PHPT should be included in the differential diagnosis of all cases of hypercalcemia. Common causes of hypercalcemia include William's syndrome, idiopathic infantile hypercalcemia, malignancy, and toxicity with drugs like thiazides and vitamin A. Rare causes include familial hypocalciuric hypercalcemia, multiple endocrine neoplasia I and II syndromes, subcutaneous fat necrosis, granulomatous disorders, hypervitaminosis D, malignancies, adrenal insufficiency [7], hyperthyroidism, hypothyroidism, and limb fracture with immobilization [2].

The laboratory diagnosis of PHPT is made by demonstrating elevated serum iPTH and calcium levels. A Sestamibi scan of the parathyroid gland can localize the overactive sites. We are presenting a unique patient with hypercalcemia due to a PA. His only symptom at presentation was frank psychosis; however, we were not able to find such a presentation of PHPT in pediatric population by a careful literature review.

## 2. Case 

Our patient is a 17-year-old African American male who was transferred to the Children's Hospital of Wisconsin (CHW) emergency room from a local inpatient psychiatric unit, for further workup of elevated serum calcium and iPTH levels. The patient had been admitted with acute psychosis a week prior to his transfer to CHW, where on a routine laboratory screening he was found to have serum calcium of 16.5 mg/dL (8.9–10.7). The serum iPTH level was markedly elevated: 315 pg/mL (9–69). During the preceding 2-3 weeks, the patient had intermittent episodes of aggressive, hostile, and angry moods associated with paranoia. He reported hearing of voices, visual hallucinations, and delusional ideation. His mother reported that he had been having difficulty initiating sleep and frequently paced in his bedroom throughout the night. He also had frequent arguments with his father and girlfriend and was reported to have suicidal and homicidal ideations. He subsequently suffered a fracture of the right radius after jumping out of a moving car. Consequently, he was admitted to an inpatient psychiatric unit with diagnosis of acute psychosis.

At the time of presentation, he was in the 12th grade with average school performance. He reported being sexually active and smoked marijuana about 2-3 times per week. He denied history of bone pain, abdominal pain, passage of red colored urine, or stones in the urine. He had a normal birth history and developmental milestones during childhood and no history of head injury. He had a history of behavioral problems since the 2nd grade and was assessed by the school social worker and the psychologist, who attributed them to underlying learning disability, and was placed in special education. He never had any prior psychiatric evaluation nor was he ever placed on psychiatric medications. His past medical and surgical history was unremarkable. There was no family history of hypercalcemia or major medical or mental illnesses.

On physical exam he appeared healthy looking, alert, oriented, and cooperative. He had normal vitals, physical exam, and speech. He appeared to be of average intelligence and moderately agitated. His psychiatric symptoms were unchanged since his first presentation in the local psychiatric hospital. He had no hyperactivity, tics, or stereotypic movements, gait was normal, and recent and remote memories were intact.

He had an elevated serum iPTH 242.0 (9–69 pg/mL) and alkaline phosphatase 156 IU/L (50–130), low serum 25 hydroxy vitamin D 18 ng/mL (20–100), and an elevated 1,25-dihydroxy vitamin D 102 pg/mL (27–71) (Quest Diagnostics, San Juan Capistrano, CA), consistent with PHPT. Thyroid hormone screening, electrolytes, urine drug screen, and vitamin A levels were normal. His 24-hour urinary calcium to creatinine ratio (mg Ca/mg Cr) was 0.60 (<0.21), and urinary calcium excretion rate was 12 mg/kg/day (<4). Phosphate/creatinine clearance ratio was 0.22 (0.02–0.22) and the percentage tubular reabsorption of phosphate (%TRP) was 78 (78–98). Urinary vanillyl mandelic acid was 2.2 mg/24 hours (<3.9) and urinary homovanillic acid was 2.2 mg/24 hours (1.4–7.2). He had a normal electrocardiogram, ultrasound, and MRI of neck and ultrasound of the kidney. A Sestamibi scan showed a positive signal just inferior to the left lobe of the thyroid gland. Multiple endocrine neoplasias type 1 (MEN-1) and MEN-2 gene analysis were negative (Mayo Medical Laboratories, Rochester, MN). Hypercalcemia was treated with intravenous fluids and calcitonin. His calcium levels were moderately decreased by intravenous calcitonin (2–4 U/kg/day); however calcitonin effect was not sustained. The patient underwent surgery for the removal of the parathyroid gland, 5 days after hospitalization (about a month after the acute onset of psychotic symptoms). Intraoperative and postoperative serum iPTH were <2.5 pg/mL, confirming the completeness of the procedure. The calcium levels gradually normalized in 48 hours (9.7 to 10.7 mg/dL), Table 1. The gland weighed 1.3 grams and its histopathology evaluation revealed a hypercellular parathyroid gland containing oxyphil and chief cells with no features of malignancy. Although his paranoid and violent behavior gradually resolved about 2-3 months after surgery, he remained under psychiatric care for behavioral issues for about 6 months after surgery.

## 3. Discussions

Psychosis may be the earliest manifestation of an endocrine disorder [8]. It has been described in patients with Cushing's syndrome [9], hyperthyroidism [10], and hypothyroidism in pediatric and adult patients [11]. It has not been associated with PHPT in the pediatric age group. Most of the available information on this subject is through the adult literature describing patients with PHPT who have presented with psychosis [12, 13], as well as with other psychiatric symptoms like delirium or dementia, depression, anxiety, lethargy or apathy, stupor, or even coma [13]. The usual symptoms are divided into three categories: (1) confusional state with clouding of consciousness, which ranged from drowsiness to stupor; (2) clear sensorium with depression, psychosis with paranoid delusions and violent or bizarre behavior; and (3) a “pseudoneurotic” form [14].

The pathogenesis of psychosis in PHPT has not been established. However, since calcium appears to play an important role in determining changes in monoamine metabolism of the central nervous system, such as modification of dopaminergic and cholinergic metabolism and release at several neuroregulatory stages, it may affect behavior and mood in some patients [15, 16]. Indeed, calcium channel blockers have been used in treating schizophrenia [16]. Prolonged periods of subclinical hypercalcemia usually precede the development of psychiatric symptoms; however there are conflicting reports regarding the relationship of the severity of the symptoms and the degree of hypercalcemia [17]. An alternate hypothesis is that the psychiatric symptoms may be related to a number of factors like premorbid adjustment and sociocultural influences [18]. In our patient, there is a history of “behavioral problems” with learning disability requiring special education since early elementary school. In adults most cases of PHPT (56%) can be asymptomatic at diagnosis [19] versus the children (16%) [20]. This may be because adults are more likely to have a screening for calcium done, although asymptomatic disease may still be common in pediatric population [21].

Depending upon the severity of hypercalcemia in PHPT, different treatment options can be utilized including hydration and by using loop diuretics like furosemide to cause calciuresis. Bisphosphonates and calcitonin have also been effective in lowering serum calcium concentrations in hypercalcemic children [5]. The other treatment options include using oral phosphate, glucocorticoids [22], and peritoneal dialysis [23]. The definitive treatment is surgery, which should be carried out as soon as possible [24]. It is effective in lowering serum calcium but may not reverse end-organ damage that has already occurred [24].

In PHPT, decreased percent tubular reabsorption of phosphate (TRP) usually results in low serum phosphate [25]; however, in some instances serum phosphate levels may fall in the low normal range as observed in our patient.Our patient's serum iPTH and calcium levels normalized after the parathyroidectomy. In addition, his serum 25-OH vitamin D levels normalized after replacement therapy.

Fujikawa et al. observed that neurobehavioral symptoms in mild primary hyperparathyroidism related to hypercalcemia might not improve after the parathyroidectomy [26]. The recovery from the psychosis secondary to the PHPT after the surgery is not related to the duration of disease, severity of changes, or the age of the patient [27].

In conclusion, we present a case of PHPT due to a parathyroid adenoma presenting with psychosis. Physicians should know that PHPT might be responsible for a wide spectrum of symptoms. Although psychotic symptoms are uncommon, as compared to affective or cognitive symptoms, they may occur as a sole manifestation of this disorder and may lead to dangerous behaviors. A screening serum calcium level should be checked in a patient presenting with psychiatric symptoms, since a delayed diagnosis can result in more severe complications. The disease is usually due to a single PA, and surgery is an extremely safe and effective method of preventing the complications of this disease and perhaps the reversal of psychiatric symptoms.

## Figures and Tables

**Table 1:** Biochemical characteristics of our patient with PHPT.

Laboratory results	BT	AMT	ASMT
Serum iCa (4.5–5.3 mg/dL)	8.0	6.6	4.8
Serum total calcium (8.9–10.7 mg/dL)	18.0	11.8	9.6
Serum iPTH (9–69 pg/mL)	350.0	242.0	<2.5
Serum 25-OH vitamin D (20–100 ng/mL)	18.0	22.0	61.0
Serum phosphate (2.5–4.8 mg/dL)	3.1	3.0	3.5
Serum alkaline phosphatase (50–130 IU/L)	156.0	143.0	122.0
Urine calcium/creatinine ratio (<0.21)	0.6	ND	ND
Urinary calcium excretion rate (<4 mg/kg/day)	12.0	ND	ND
Phosphate/creatinine clearance ratio (0.02–0.22)	0.22	ND	ND
Tubular reabsorption of phosphate (TRP) (78–98)%	78.0	ND	ND

BT: before treatment; AMT: after medical treatment; ASMT: after surgery and medical treatment; ND: not done.
